# Inhaled nitric oxide suppresses neuroinflammation in experimental ischemic stroke

**DOI:** 10.1186/s12974-023-02988-3

**Published:** 2023-12-15

**Authors:** Rebecca I. Sienel, Uta Mamrak, Janina Biller, Stefan Roth, Andreas Zellner, Tipparat Parakaw, Rayomand S. Khambata, Arthur Liesz, Christof Haffner, Amrita Ahluwalia, Burcu F. Seker, Nikolaus Plesnila

**Affiliations:** 1 Institute for Stroke and Dementia Research, Klinikum der Universität München and Ludwig Maximilian University (LMU) Munich, Feodor-Lynen Str. 17, 81377 Munich, Germany; 2 Munich Cluster for Systems Neurology (SyNergy), Munich, Germany; 3 William Harvey Research Institute, Barts & The London School of Medicine and Dentistry, Queen Mary University of London, London, UK

## Abstract

Ischemic stroke is a major global health issue and characterized by acute vascular dysfunction and subsequent neuroinflammation. However, the relationship between these processes remains elusive. In the current study, we investigated whether alleviating vascular dysfunction by restoring vascular nitric oxide (NO) reduces post-stroke inflammation. Mice were subjected to experimental stroke and received inhaled NO (iNO; 50 ppm) after reperfusion. iNO normalized vascular cyclic guanosine monophosphate (cGMP) levels, reduced the elevated expression of intercellular adhesion molecule-1 (ICAM-1), and returned leukocyte adhesion to baseline levels. Reduction of vascular pathology significantly reduced the inflammatory cytokines interleukin-1β (Il-1β), interleukin-6 (Il-6), and tumor necrosis factor-α (TNF-α), within the brain parenchyma. These findings suggest that vascular dysfunction is responsible for leukocyte adhesion and that these processes drive parenchymal inflammation. Reversing vascular dysfunction may therefore emerge as a novel approach to diminish neuroinflammation after ischemic stroke and possibly other ischemic disorders.

**Supplementary Information:**

The online version contains supplementary material available at 10.1186/s12974-023-02988-3.

## Introduction

Each year, approximately 12.2 million people worldwide experience a stroke [1]. Current therapeutic approaches include thrombolysis and endovascular thrombectomy; however, due to their high level of complexity—even in industrialized countries—only 15% of patients undergo these procedures [2]. Moreover, although recanalization can successfully restore cerebral blood flow, oxygen supply, and metabolic substrates to post-ischemic tissues, they do not prevent secondary inflammatory events, which may persist even after reperfusion.

Post-ischemic neuroinflammation plays a substantial role in the pathophysiology of ischemic stroke [3]. Emerging experimental evidence and clinical studies show that the immune system is intimately involved in many stages of the ischemic pathological cascade by activating both innate and adaptive immune responses [4]. This process eventually results in the production of inflammatory cytokines and an upregulation of adhesion molecules, promoting the infiltration of leukocytes into the brain [5–7]. This notion is supported by studies showing that depleting leukocytes and/or inhibiting the adhesion of leukocytes to the cerebral endothelium have neuroprotective effects [8–10]. Unfortunately, however, subsequently developed immunomodulatory therapies such as pharmacologically blocking either cytokine receptors or leukocyte adhesion molecules were not successful in clinical trials [11]. Thus, new, effective anti-inflammatory approaches are urgently needed in order to improve patient outcome.

The signaling molecule nitric oxide (NO) has a wide range of key functions in the body, ranging from controlling cellular homeostasis to regulating neural, endothelial, and immune processes [12, 13]. Following ischemic stroke, a genetically induced increase in vascular NO has been shown to confer a strong neuroprotective effect in animal models [14, 15]; however, systemic application of an NO donor acutely reduce blood pressure, making this therapy undesirable for use in patients following a stroke [16]. To overcome these side effects associated with systemic NO donors, inhaled NO (iNO) may be a clinically acceptable alternative; indeed, iNO has been shown previously to have a protective effect in animal models of ischemic stroke and cardiac ischemia [17–19]. However, the underlying mechanism is currently unknown.

Here, we examined whether iNO can exert anti-inflammatory effects following experimental stroke. Specifically, we examined whether iNO increases the bioavailability of systemic NO, whether iNO affects the leukocyte–endothelium interaction, and how iNO affects the molecular mechanisms involved in the adhesion of leukocytes to the cerebrovascular endothelium.

## Results

### Nitrite and nitrate levels in blood, urine, and brain after iNO

**Fig. 1:**
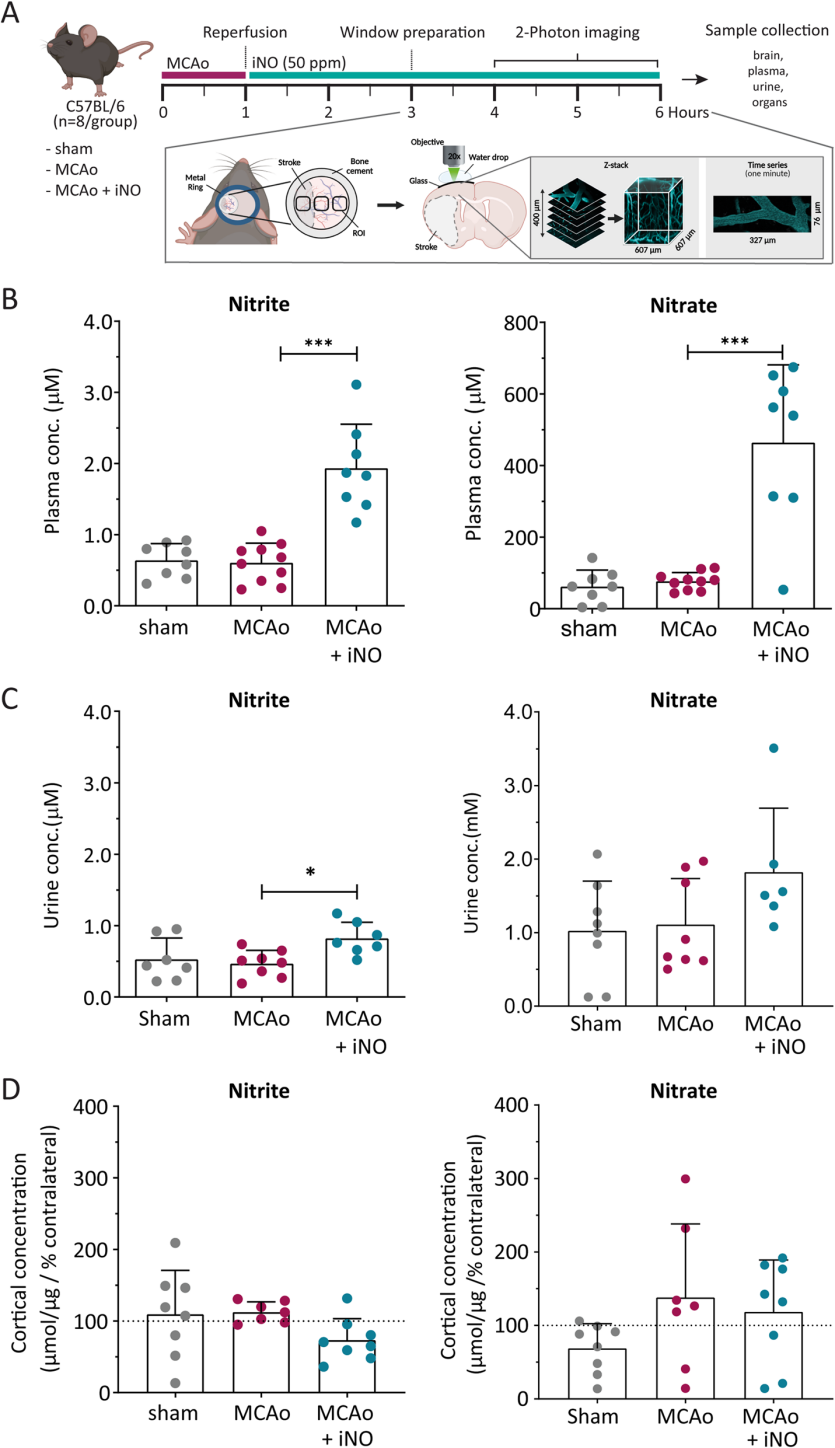
Experimental protocol and plasma, urine, and cortical nitrite and nitrate levels. **A** Time course depicting the protocol for inducing acute transient cerebral ischemia. Quantification of nitrite and nitrate concentrations measured in plasma (**B**), urine (**C**), and brain (**D**) samples collected from the indicated groups. *p < 0.05 and ***p < 0.001; one-way ANOVA (**B**–**D**) or Kruskal–Wallis test p: 0.217 (**C**: Urine nitrate); n = 7–10 per group

Nitrite and nitrate, which serve as storage forms of NO in mammals [20], were measured in plasma and brain homogenates 6 h after stroke induction using a chemiluminescence assay. As expected, we found that MCAo alone had no effect on plasma nitrite or nitrate levels compared to sham operated mice; in contrast, both nitrite and nitrate levels were significantly increased in the iNO-treated MCAo group (Fig. 1B). Excess NO metabolites are eliminated via renal excretion, and we found a slight but significant increase in urine nitrite levels in the iNO-treated group compared to the untreated MCAo group (Fig. 1C). Finally, we found no significant difference between groups with respect to the cortical levels of either nitrite or nitrate (Fig. 1D).

### Inhaled NO modulates downstream signaling molecules involved in leukocyte adhesion

**Fig. 2:**
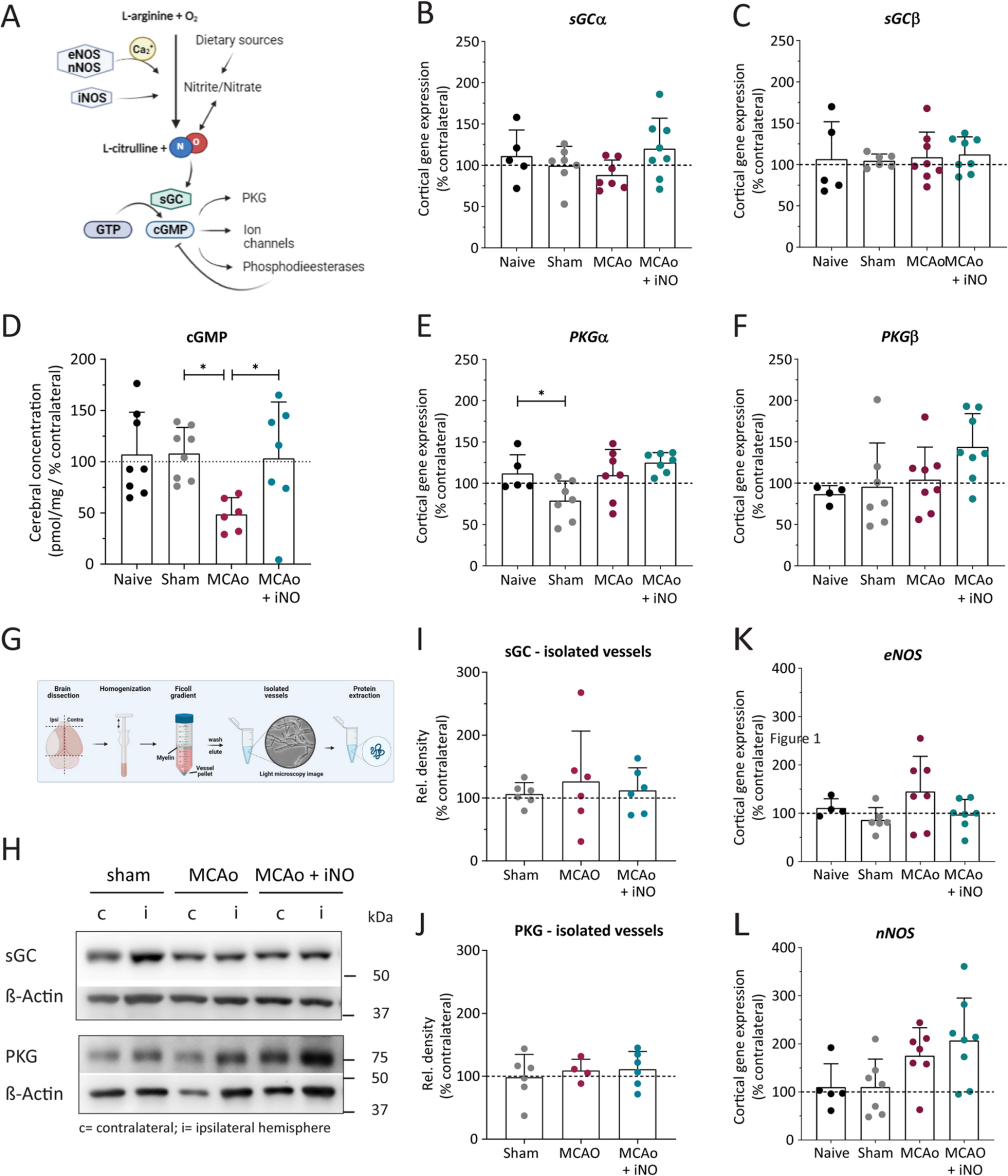
Effect of iNO on downstream signaling molecules. **A** Illustration depicting the NO signaling pathway. **B**–**F** Quantification of sGCα mRNA (**B**), sGCβ mRNA (**C**), cGMP levels (**D**), PKGα mRNA (**E**), and PKGβ mRNA (**F**) measured in the ipsilateral hemisphere in the indicated mice, expressed relative to the contralateral hemisphere. **G** Illustration depicting the strategy used to isolate and purify cerebral vessels (created using BioRender). **H**–**J** Representative western blot (**H**) and quantification (**I** and **J**) of the indicated proteins measured in vessels isolated from the indicated groups (c, contralateral; i, ipsilateral). **K**, **L** Quantification of eNOS and nNOS mRNA levels measured in the ipsilateral cerebral cortex in the indicated groups, expressed relative to the contralateral hemisphere. *p < 0.05 one-way ANOVA; n = 4–8 per group

To investigate the role of inhaled NO on downstream signaling involved in leukocyte–endothelium interaction following cerebral ischemia, we collected the brain 6 h after stroke induction. We measured endothelial NO synthase (eNOS), neuronal NO synthase (nNOS), inducible NO synthase (iNOS), the NO receptor sGC (soluble guanylyl cyclase), the sGC product cGMP, and cGMP-dependent protein kinase (PKG) levels in brain lysates and/or isolated cerebral vessels (Fig. 2A). We found no significant difference between groups with respect to the cortical mRNA levels of either the sGCα (Fig. 2B) or sGCβ (Fig. 2C) subunit. In contrast, cortical cGMP levels were significantly reduced in the MCAo group, and this reduction was prevented by iNO treatment (Fig. 2D). Finally, we measured a slight but significant decrease in cortical PKGα expression in the sham-operated mice compared to naïve mice, but no other significant differences in either PKGα (Fig. 2E) or PKGβ expression between groups (Fig. 2F).

Next, we isolated cerebral vessels and measured the effects of iNO treatment on sGC and PKG protein levels following MCAo (Fig. 2G). Using western blot analysis (Fig. 2H), we found no significant difference between groups with respect to sGC (Fig. 2I) or PKG (Fig. 2J) protein levels. Thus, the application of inhaled NO had no effect on the expression of sGC or PKG following MCAo.

Lastly, we found no significant difference between groups with respect to either *eNOS* (Fig. 2K) or *nNOS* (Fig. 2L) mRNA levels measured in the cerebral cortex. We were unable to detect the expression of iNOS in any group (data not shown). Thus, inhaled NO did not affect the expression of proteins involved in NO-sGC-cGMP signaling but restored the ischemia-induced reduction of the endothelial signaling molecule cGMP.

### Inhaled NO reduces leukocyte–endothelium interactions in vivo

**Fig. 3:**
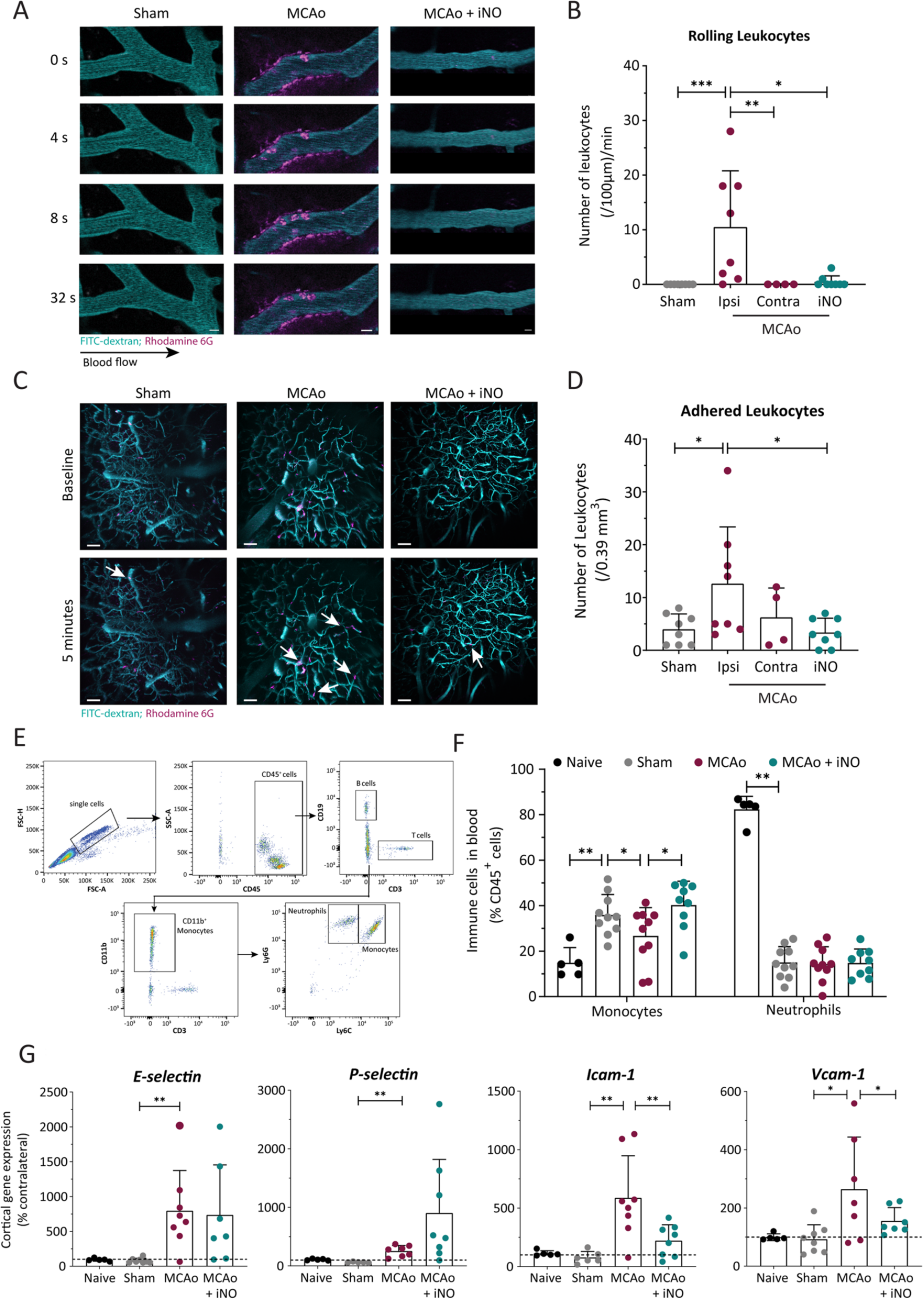
Leukocyte–endothelium interactions measured in vivo increased following stroke and were reduced by iNO treatment. **A** Example images of rolling leukocytes along the wall of cerebral venules measured 4 h after sham surgery (left), MCAo (center), and MCAo followed by iNO treatment (right); leukocytes and vessels were stained with rhodamine 6G (magenta) and FITC-dextran (cyan), respectively. The direction of blood flow is indicated by the arrow, and scale bars represent 10 μm. **B** Summary of the number of rolling leukocytes measured in the cerebrovascular endothelium. **C** Representative images of adhered leukocytes (magenta) and the cerebral vasculature (cyan); arrows indicate stalled leukocytes, and scale bars represent 50 μm. **D** Quantification of the total number of leukocytes adhered to the endothelium in the indicated groups. **E** Flow cytometry gating strategy used to analyze the immune cells in the blood samples. **F** Quantification of Ly6G-high (neutrophils) and Ly6C-high (monocytes) cells in the indicated groups. **G** The mRNA levels of the indicated selectins and integrin ligands were measured in the ipsilateral cerebral cortex in the indicated groups and are expressed relative to the corresponding contralateral hemisphere. *p < 0.05, **p < 0.01, and ***p < 0.001; Kruskal–Wallis (**B** p: 0.0006, **F**: p: 0.006 (Neutrophils) and **G** p: 0.0017 and 0.0005 (selectins)) or one-way ANOVA (**D**, **F** (Monocytes) and **G** (integrin ligands)); n = 5–10 per group

To measure leukocyte–endothelium interactions in the cerebral cortex 3 h after reperfusion following 60 min of middle cerebral artery occlusion (MCAo), the cerebral vessels were stained with FITC-dextran (to visualize the vessel lumen) and rhodamine 6G (to visualize leukocytes), and in vivo 2-photon microscopy was performed for 2 h starting 4 h after MCA occlusion (Additional file 1: Fig. S1A). Consistent with a neuroinflammatory response, we found large numbers of rolling leukocytes in the ipsilateral hemisphere of mice in the MCAo group, but not in the contralateral hemisphere or sham-operated mice (Fig. 3A, B). In contrast, virtually no rolling leukocytes were observed in the iNO-treated MCAo group (Fig. 3C, D).

After tracking rolling leukocytes along the vessel wall, we then identified immune cells that were firmly adhered to the endothelium, defined here as "adherent" when they were stationary for at least 5 min (Fig. 3C); we then counted the number of adherent leukocytes in a volume of interest of 0.39 mm^3^. We found that the sham-operated animals had an average of approximately 4 leukocytes/0.39 mm^3^, and this number increased significantly (to approximately 12 leukocytes/0.39 mm^3^) in the MCAo group, but was reduced to baseline levels in the iNO-treated MCAo group (Fig. 3D). As an internal control, the contralateral hemisphere in the MCAo group had approximately 6 leukocytes/0.39 mm^3^ (not significantly different from the ipsilateral hemisphere), confirming that inducing stroke does not affect leukocyte adhesion in the contralateral hemisphere (Fig. 3D).

### iNO alters the profile of circulating leukocytes following stroke

Next, we examined the profile of circulating leukocytes by collecting whole blood samples and performing FACS analysis (Fig. 3E). We found that sham-operated animals had significantly increased numbers of circulating monocytes and neutrophils compared to control (i.e., naïve) animals (Fig. 3F). Following MCAo, the number of monocytes decreased, while neutrophils were unchanged, and this reduction in circulating monocytes was prevented in iNO-treated animals (Fig. 3F).

### iNO decreases the expression of adhesion molecules in the brain following stroke

Given that iNO decreased the interaction between leukocytes and the cerebrovascular endothelium and restored the number of circulating immune cells, we examined the effect of iNO on the expression of adhesion molecules. Specifically, we measured the mRNA levels of the genes expressing E-selectin (*SELE*), P-selectin (*SELP*), intercellular adhesion molecule 1 (*ICAM1*), and vascular cell adhesion molecule 1 (*VCAM1*) in the brain using qPCR; we examined these specific selectins and integrin ligands because these molecules mediate the rolling and firm adhesion of immune cells to the vascular wall of post capillary venules. We found that the mRNA levels of all four adhesion molecules were significantly increased 5 h after stroke induction compared to both naïve and sham-operated mice (Fig. 3G). Interestingly, iNO treatment significantly reduced the expression of *ICAM1 and VCAM1* (Fig. 3G), the main adhesion molecules responsible for the firm adhesion of leukocytes to the vascular endothelium, but did not significantly affect the other two adhesion molecules (Fig. 3G).

**Fig. 4:**
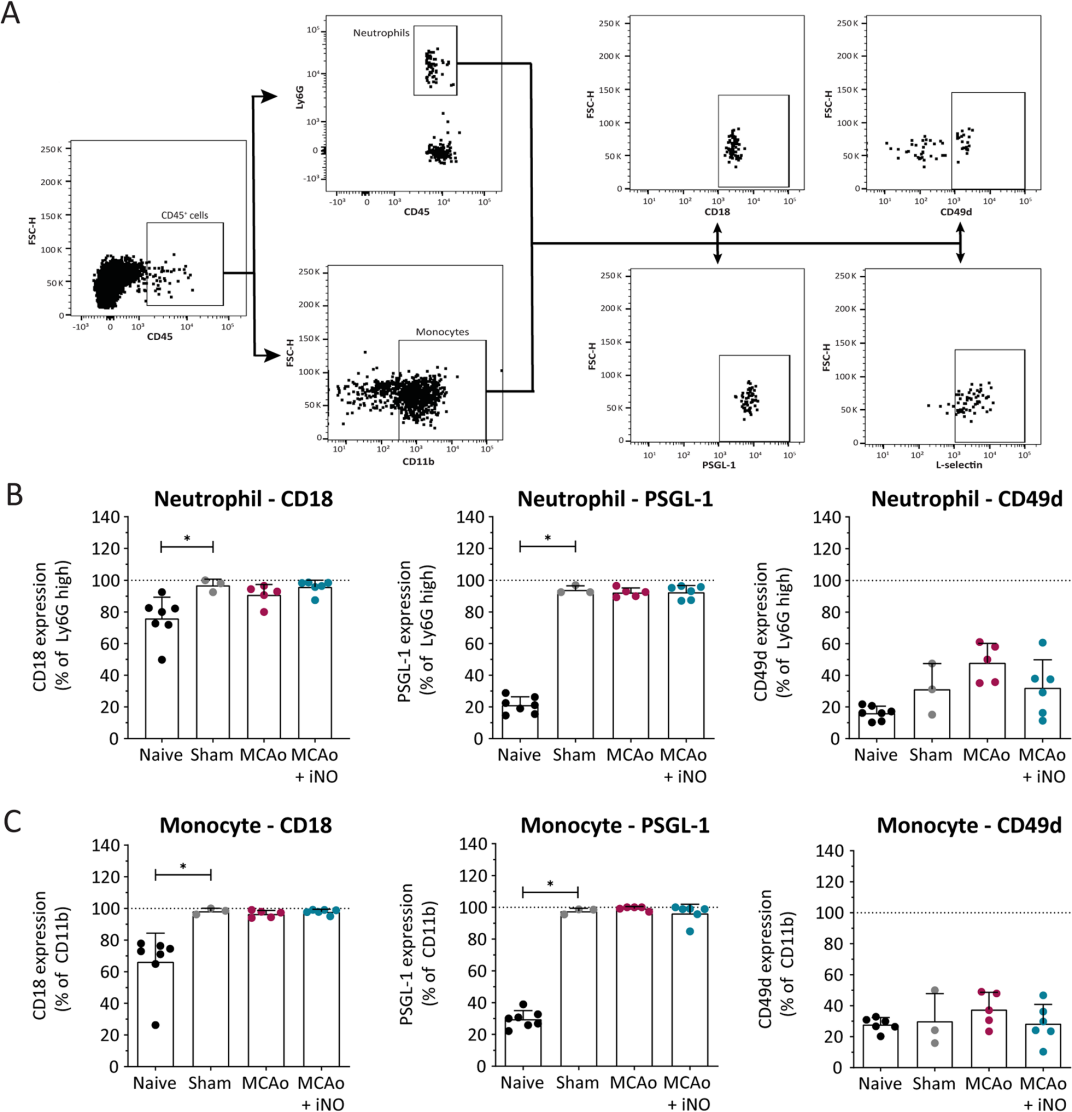
Adhesion molecule markers measured on circulating leukocytes. **A** The gating strategy used for flow cytometry. **B**, **C** Quantification of Ly6G-high (neutrophils; **B**) and CD11b-high (monocytes; **C**) cells and the indicated surface adhesion molecules measured in the indicated groups. *p < 0.05; one-way ANOVA; n = 3–7 per group

In addition, we examined the counter ligands of endothelial adhesion molecules expressed on circulating immune cells by isolating myeloid cells from the peripheral blood and performing flow cytometry (Fig. 4A). We found that sham surgery significantly up-regulated both CD18 and PSGL-1 expression in neutrophils (Fig. 4B) and monocytes (Fig. 4C) compared to naïve mice, and both MCAo alone and iNO treatment in MCAo mice had similar effects as sham surgery. Finally, we found that CD49d expression was similar between all four experimental groups (Fig. 4B, C).

### iNO reduces cerebral cytokine levels following stroke

**Fig. 5:**
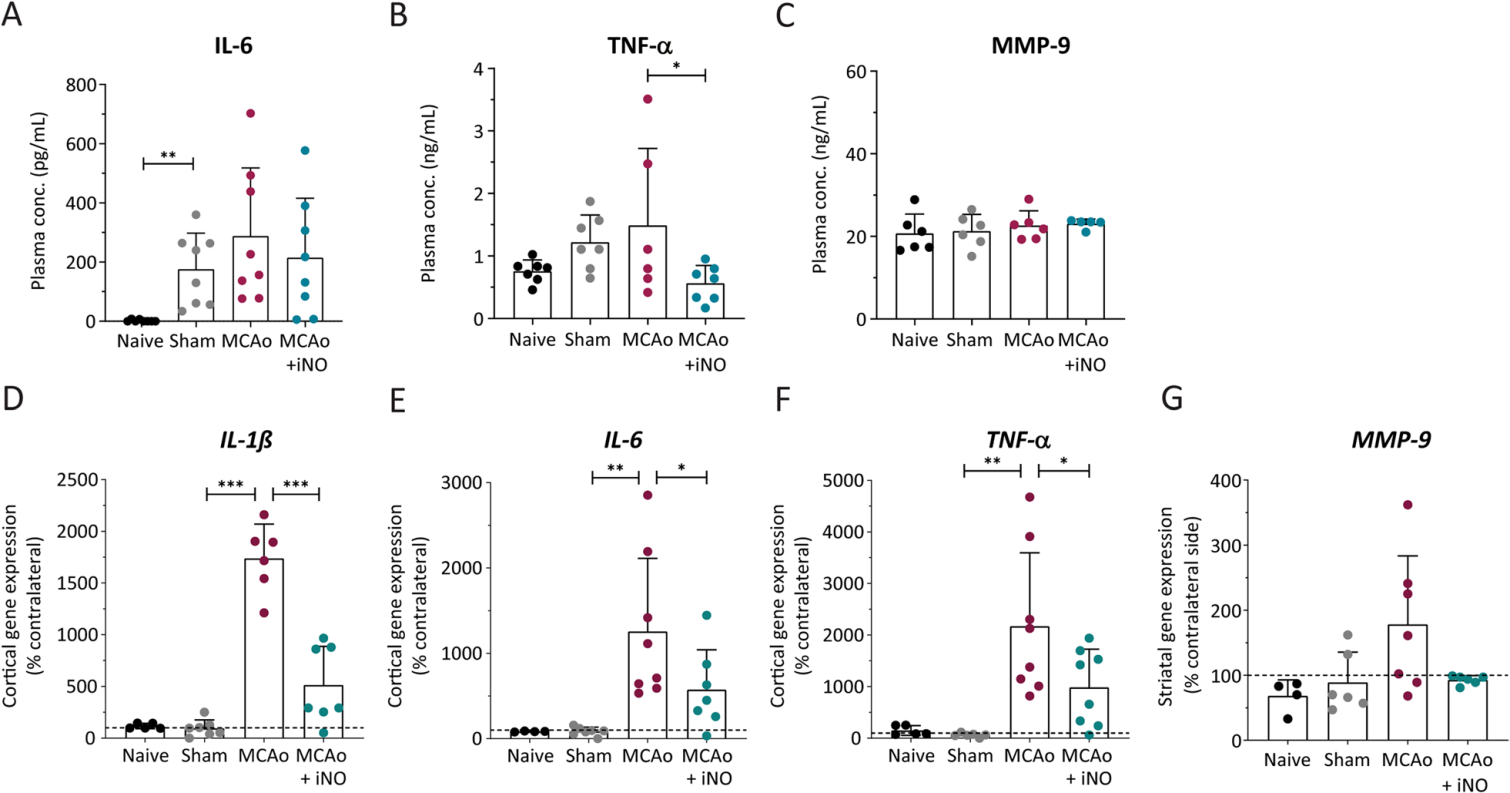
Pro**-**inflammatory cytokines increased in the cortex following MCAo and were decreased by iNO treatment. **A**–**C** Quantification of IL-6, TNF-α, and MMP-9 levels measured in the plasma using ELISA. **D**–**G** Quantification of IL-1β, IL-6, TNF-α, and MMP-9 mRNA measured in the ipsilateral hemisphere in the indicated groups, expressed relative to the corresponding contralateral hemisphere. *p < 0.05, **p < 0.01, and ***p < 0.001; Kruskal–Wallis p: 0.0006 (**A**) or one-way ANOVA (**B**–**G**); n = 4–8 per group

To further investigate the putative anti-inflammatory properties of iNO following stroke, we measured the expression of the pro-inflammatory cytokines interleukin 6 (IL-6), interleukin 1β (IL-1β), and tumor necrosis factor-alpha (TNF-α) in brain tissue and IL-6 and TNF-α in peripheral blood 5 h after reperfusion. Due to limited sample volume Il-1ß was not measured in plasma. We found that the plasma levels of IL-6 were significantly higher in sham-operated mice compared to naïve mice, and neither MCAo alone nor iNO treatment significantly affected these levels (Fig. 5A). With respect to plasma TNF-α levels, we found similar levels between naïve, sham-operated, and MCAo mice, with iNO treatment significantly reducing plasma TNF-α levels in MCAo mice (Fig. 5B).

The matrix metalloproteinase family member MMP-9 plays an important role in disrupting the blood–brain barrier following ischemia. Therefore, we measured MMP-9 in the plasma 5 h after reperfusion and found no significant difference between groups with respect to plasma MMP-9 levels (Fig. 5C).

In contrast to our results regarding pro-inflammatory cytokines in the circulation, we found that cerebral ischemia significantly up-regulated the expression of IL-1β (Fig. 5D), TNF-α (Fig. 5F), and IL-6 (Fig. 5E) mRNA in cortical tissue. This upregulation was significantly reduced by iNO-treatment (Fig. 5D–F).

Finally, consistent with our results on plasma MMP-9, we found no significant difference between groups with respect to MMP-9 expression measured in the cortex (Fig. 5G).

## Discussion

The pathophysiology of stroke is complex and remains poorly understood. However, both preclinical and clinical studies have shown that stroke can elicit a robust neuroinflammatory response. Although this neuroinflammation is essential for the healing and repair process occurring days and weeks after stroke, it can cause additional brain damage within the first hours following stroke. Thus, therapeutic strategies that target neuroinflammation must be designed and applied with care [21].

A wealth of experimental data suggests that targeting neuroinflammation within the first few hours following the onset of ischemic stroke may represent a promising therapeutic strategy [11, 22]. To test this hypothesis, we investigated the anti-inflammatory effects of administering NO by inhalation immediately following experimental stroke. The effects of NO on the autoregulation of cerebral blood flow are well known, and a lack of NO following cerebral ischemia has been shown to cause vasoconstriction and additional brain damage [17, 23–26]. Indeed, NO was previously delivered by inhalation to avoid systemic side effects and was shown to restore vascular function in both small-animal and large-animal models of ischemic stroke and brain trauma [17, 27–29]. In addition to its vasoactive properties, NO also has anti-adhesion properties [18, 30–32]; thus, inhaled NO may reduce the interaction between leukocytes and the cerebrovascular endothelium, thereby providing neuroprotection following focal cerebral ischemia. We used deep brain in vivo 2-photon microscopy to visualize the early phases of post-stroke inflammation (specifically, 4 h after a 1-h MCA occlusion followed by reperfusion). Our results show that iNO potently reduced leukocyte rolling and adhesion to baseline levels without causing any apparent systemic side effects (Additional file 2: Fig. S2). Nevertheless, potential side effects of iNO should be considered; we therefore delivered a relatively low concentration of NO by inhalation (50 ppm) together with physiologically relevant oxygen concentrations, as reported by other groups [33–35].

NO produced by endothelial cells has a well-known antiadhesive effect on circulating blood leukocytes and platelets, which is most likely, mediated by the suppression of adhesion molecules by continuous NO-mediated signaling through the cGMP/PKA [36–38]. Since cerebral ischemia reduces cerebral cGMP and iNO reverses this process, we assume that the molecular mechanisms responsible for the anti-inflammatory effect of iNO is most likely mediated by reinitiating cGMP/PKA signaling.

By measuring the expression of several adhesion proteins, we found that iNO reduced *ICAM1* mRNA levels in the cerebral cortex following MCAo, consistent with reports by other groups using other disease models and peripheral organs [38–41]. This reduction of adhesion proteins may provide a plausible explanation for how iNO prevents the interaction between circulating leukocytes and the cerebrovascular endothelium, thereby reducing the transmigration of leukocytes into the ischemic brain tissue. In contrast to its effects on the expression of ICAM-1 and VCAM-1 molecules that promote firm adhesion—iNO had no apparent effect on E-selectin or P-selectin expression following cerebral ischemia. This finding is in contrast with other studies that found that NO caused a decrease in selectins [38, 41, 42]. These apparent discrepancies may be due to differences in the timing and/or duration of the treatment, as well as the animals’ physiological condition [38, 41, 42].

Another putative regulatory target of NO is the expression of adhesion molecules on leukocytes, which serve as the counterpart to the adhesion molecules expressed on endothelial cells. We found that iNO caused a slight—albeit not significant—decrease in CD49d (the counterpart to VCAM-1) expression in both neutrophils and monocytes in mice following MCAo; in contrast, iNO had virtually no effect on CD18 (the counterpart to ICAM-1) expression. Although previous studies suggest that NO can inhibit CD18 [43–45], the mechanism of action is currently unknown. Interestingly, Banick et al. suggested that NO inhibits CD18 on neutrophils by affecting the activity of guanylyl cyclase at the cell membrane [44]. This would in turn inhibit the conformational change in the CD18 molecule and prevent its binding to ICAM-1 on the endothelium. Moreover, two additional studies suggest that a multifaceted series of events mediate inhibition via the cytoskeleton in neutrophils [45, 46]. Thus, despite previously reported findings, the adhesion molecules expressed on leukocytes do not appear to be the primary target mediating the observed anti-adhesion effects of iNO, and further research is warranted in order to investigate this issue in more detail.

In addition to regulating the leukocyte–endothelium interaction, NO is a potent endogenous inhibitor of pro-inflammatory cytokines, molecules that up-regulate the expression of cell adhesion molecules and attract phagocytic cells [38, 47]. Three major pro-inflammatory cytokines—IL-1β, TNF-α, and IL-6—both mediate and aggravate the inflammatory response following stroke [48]. We found that all three of these pro-inflammatory cytokines were up-regulated in the ischemic cortex, consistent with previous studies suggesting a connection between these pro-inflammatory cytokines and infarct size in experimental stroke [49]. We also found that iNO significantly reduced the increase in these three cytokines in the affected hemisphere. However, studies have shown that in addition to causing a local increase in cytokines, stroke also increases systemic cytokine levels. For example, Clausen et al. recently reported that cytokine levels are increased in both the cerebrospinal fluid and peripheral blood in patients following ischemic stroke [50]. Therefore, cytokines—particularly TNF, IL-1, and IL-6—have attracted considerable interest as potential markers for stroke severity and neurological outcome [51, 52]. In our study, we observed a slight—albeit not significant—increase in plasma IL-6 and TNF-α levels in mice following MCAo, and iNO was more effective at reducing TNF-α levels compared to IL-6 levels. Thus, it is reasonable to speculate that iNO may either reduce the production of cytokines in the ischemic tissue thereby reducing their spill over into the systemic circulation or may act at both local and systemic levels to reduce inflammation. In any case, inhaled NO significantly reduces brain and plasma cytokine levels following ischemic stroke and may thereby prevent further brain damage.

Previous studies involving peripheral tissues have shown that reducing NO levels by pharmacologically blocking NO production or genetically ablating NOS enzymes induces leukocyte adhesion [18, 53–55]. Conversely, restoring NO availability was shown to reduce leukocyte adhesion in various organs in the context of ischemia/reperfusion injury [18, 32]. Extending these experimental results to a clinical context has confirmed the anti-inflammatory potential of NO; however, the negative systemic effects of administering NO limit the clinical value of these therapies. Therefore, the delivery of NO via inhalation is a promising alternative, as it can potentially avoid the effects associated with systemic NO administration and can restrict the effects of NO to the affected area. Outside the brain the favorable anti-inflammatory effects of iNO have already been shown in clinical settings, for example following knee surgery and liver and heart transplantation [30–32]. Under these conditions, the anti-adhesion effects of NO were seen predominantly in the capillaries and venules, however, as hypoperfusion shifts hemoglobin deoxygenation towards the arterioles, NO may also inhibit leukocyte–endothelium interactions in these vessels [56, 57].

Delivering NO application via the pulmonary circulation raises the question of whether the NO reaches the desired location (in this case, the brain). However, the vascular effects of inhaled NO have been shown to extend beyond the pulmonary circulation via the formation of NO carriers such as nitrite, nitrate, and S-nitrosothiols (e.g., hemoglobin, cysteine, glutathione, and albumin) [18, 20, 58]. Here, we report that plasma nitrite and nitrate levels were significantly higher in iNO-treated mice, suggesting that these metabolites potentially serve as the principal transporters of NO. This notion is supported by other studies using iNO and suggests that these intermediates—either individually or together—may contribute to the putative neuroprotective effects of NO [59, 60]. In the presence of oxygenated hemoglobin, nitrite is rapidly oxidized to form nitrates [61], potentially explaining the larger increase in plasma nitrate levels seen in our study. In the presence of reduced tissue oxygenation and acidosis that occur in cerebral ischemia, NO is produced locally and released directly from heme groups, along with a reduction in oxygen, S-nitroso-albumin, and/or from nitrite by the activity of nitrite reductases including potentially deoxyhaemoglobin and xanthine oxidoreductase [58, 62]. Thus, NO delivered via inhalation can exert its effects as a vasodilator beyond the pulmonary circulation.

We also investigated the effects of NO on downstream signaling in the brain and found that iNO slightly—albeit not significantly—increased the expression of sGCα. Thus, it is reasonable to speculate that inhaled NO increases the expression of the principal cGMP-producing enzyme that has reduced function following stroke due a reduction in the sGCα subunit [63, 64]. By helping to maintain sGC (GC-1) expression, iNO treatment was also able to increase cortical cGMP levels following stroke. Together, our findings suggest that iNO treatment reduces the expression of pro-inflammatory adhesion molecules and helps restore neuroprotective signaling via the sGC-cGMP signaling pathway.

Finally, we also examined the expression of several NO synthases, as the application of iNO may have affected endogenous NO levels by modulating these enzymes. We found that MCAo either alone or followed by iNO treatment had no significant effect on either eNOS or nNOS expression. Under physiological conditions, NO produced by eNOS localized in the Golgi apparatus and neighboring caveolae suppresses inflammatory activation of the endothelium [65]. Thus, the activation of inflammatory mediators during ischemia may up-regulate eNOS [66, 67].

A secondary observation of our study is that a surgical intervention alone can induce a pronounced immune response. For example, we found that mice that underwent sham surgery at the neck (without occlusion of the MCA) displayed an increased proportion of circulating neutrophils and monocytes and substantially increased the expression of adhesion molecules on their surface compared to naïve animals. The increased number of peripheral monocytes is likely derived from the spleen, an immediate reservoir for monocytes [68]. This observation was only possible because we used unhandled (naïve) mice as controls. These results clearly indicate that appropriate controls are indispensable when investigating peripheral immune responses in animal models which require surgery.

In summary, a growing body of evidence suggests that inflammatory processes contribute to the formation of injury following cerebral ischemia. Here, we present evidence that NO delivered via inhalation, that is via the pulmonary system, inhibits leukocyte adhesion and suppresses pro-inflammatory signaling in the brain parenchyma, in addition to its vasoactive properties. Thus, iNO, which is clinically approved for the treatment of acute respiratory failure and pulmonary hypertension, positively modulates several stroke-related pathophysiological pathways and may represent a novel and potentially safe therapeutic strategy for use in patients following stroke.

## Materials and methods

### Animals

Male C57BL/6 mice (6–8 weeks, 24–26 g; Charles River Laboratories, Sulzfeld, Germany) were used for this study. All animals had free access to tap water and pellet food, and all experiments were conducted in accordance with institutional guidelines approved by the government of Upper Bavaria (license number 17-152). The animals were randomly assigned to the various experimental groups (sham, MCAo, and MCAo + iNO), and the data were analyzed by researchers who were blind with respect to the treatment groups. In this study two cohorts of animals were investigated: one received cranial windows and was used for intravital microscopy and a second one did not receive cranial windows and was used for biochemical analysis.

### Transient focal cerebral ischemia

To induce focal ischemia, the left middle cerebral artery (MCA) was transiently occluded as described previously [5–7]. In brief, the mice were anesthetized with 2% isoflurane in air supplemented with oxygen (30%) delivered via a nasal mask for the duration of the surgery (< 25 min), during which the animal’s body temperature was maintained at 37 ± 0.1 °C using a feedback-controlled heating pad (FHC, Bowdoinham, ME). Focal cerebral ischemia was induced by occluding the MCA with a silicone-coated filament (Doccol Corporation, Sharon, MA; catalog #701912PK5Re). Complete occlusion of the MCA was confirmed by measuring regional cerebral blood flow (rCBF) in the affected area using a PeriFlux System 5000 laser Doppler fluxmeter (Perimed, Järfälla, Sweden). The animal was subsequently re-anesthetized with isoflurane, the filament was removed 60 min after insertion to allow reperfusion, and the wound was subsequently closed. Sham-operated animals underwent the same surgical procedure, but without vessel occlusion.

### Nitric oxide (NO) inhalation

Directly after reperfusion, the animals were placed in a custom-made air-tight box and exposed to ambient air supplemented with 50 ppm NO (268 mg/m^3^ N_2_; Linde, Dublin, Ireland) for 2 h. The concentrations of NO and N_2_O were monitored continuously using a gas detector (Industrial Scientific, Pittsburgh, PA). Thereafter, animals were anesthetized by an intraperitoneal (i.p.) injection of medetomidine (0.5 mg/kg), midazolam (5 mg/kg), and fentanyl (0.05 mg/kg), intubated, and mechanically ventilated (MiniVent Type 845 Hugo Sachs Elektronik, March, Germany) with oxygen-enriched air (50% O_2_) supplemented with 50 ppm NO for four additional hours while preparing the cranial window for intravital microscopy.

### Intravital microscopy

An acute cranial window was prepared over the MCA area, and the fluorescent dyes FITC-dextran and rhodamine 6G (0.5% in phosphate-buffered saline (v/v); Sigma-Aldrich, St. Louis, MO) were injected via an arterial catheter to visualize the vasculature and leukocytes, respectively. The mouse was then placed under a 2-photon microscope (LSM 7 MP Zeiss, Oberkochen, Germany) equipped with a Li:Ti laser (Coherent, Inc., Santa Clara, CA) and fixed using a head ring and a palate plate holder (David Kopf Instruments, Tujunga, CA). A 20 × water-immersion objective (Plan Apochromat, NA 1.0; Zeiss, Oberkochen, Germany) was used for imaging. To measure adhesion, *Z*-stacks (607.28 × 607.28 µm; 512 × 512 pixels) at a 1-µm step distance were collected to a depth of 400 µm and later combined to generate a three-dimensional reconstruction. Time series (1 min duration) images 327 × 76.64 µm (512 × 120 pixels) in size were also recorded to measure the properties of rolling immune cells. Randomly selected segments (20–50 µm in length) of pial arteries were scanned 1000 times at maximum speed (in line scan mode) and used to calculate of the moving erythrocytes (to measure blood flow velocity). Physiological parameters were monitored throughout the entire procedure (Additional file 2: Fig. S2).

### Isolation of cerebral vessels

Frozen brain hemispheres were used to isolate cerebral vessels. Specifically, the tissues were cut into small pieces and homogenized using a glass tissue grinder (DWK Life Sciences, Wertheim, Germany) in 15 mL cold minimum essential medium (MEM, Thermo Fisher Scientific, Waltham, MA). The homogenate was mixed with Ficoll (Sigma-Aldrich; catalog #F4375-500G) to a final concentration of 15%, and then centrifuged at 6000×*g* for 20 min at 4 °C. The supernatant was discarded, and the pellet was suspended in 1% (w/v) bovine serum albumin (Sigma-Aldrich) in cold PBS, transferred onto a 40-µm nylon mesh (Becton Dickinson, Franklin Lakes, NJ), and washed thoroughly with cold PBS. The purified vessels were collected by washing the inverted nylon mesh with PBS and pelleted by centrifugation at 3000×*g* for 5 min. The purity of the resulting vessels was confirmed using an Eclipse TS 100 light microscope (Nikon, Tokyo, Japan).

### Protein extraction and Western blot analysis

To extract protein from isolated cerebral vessel a SDT lysis buffer (4% (w/v) SDS, 100 mM Tris–HCl pH 7.6, 100 mM DTT) was used. Samples were incubated for 30 min at room temperature and afterwards homogenized by Precellys tissue homogenizer (5 × 30 s, 10,000 rpm, 30 s pause). Homogenates were heated for 5 min at 95 °C and subsequently sonicated with a VialTweeter sonicator (5 times, 30 s, amplitude 100%, duty cycle 50%) (Hielscher, Teltow, Germany). A centrifugation step at 18,000×*g* for 30 min at 15 °C followed. The supernatant was collected and protein concentration determined using the colorimetric 660-nm assay according to the manufacturer’s instructions (Thermo Fisher Scientific). Protein lysates were analyzed by sodium dodecyl sulfate–polyacrylamide gel electrophoresis and electrotransfer onto 0.2-µm nitrocellulose membranes using the Mini-Protean and Trans-Blot system (Bio-Rad Laboratories, Hercules, CA, USA). Membranes were blocked with 5% skim milk powder dissolved in Tris-buffered saline supplemented with 0.1% Tween (TBS-T) for 1 h at RT and then incubated with anti-ICAM-1 (ab222736, Abcam 1:1000), anti-PKG-1 (#3248, Cell Signaling, 1:1000), or anti- Guanylate Cyclase β1 subunit (#160897, Caymanchem, 1:200) primary antibody (diluted in blocking buffer) overnight at 4 °C. Subsequently, blots were washed and probed with horseradish peroxidase-conjugated anti-rabbit (Thermo) secondary antibody diluted 1:7500 in blocking buffer for 1 h at RT. Immuno-reactive bands were visualized using chemiluminescence development (Immobilon ECL detection reagent, Merck Millipore) and the Fusion FX7 imaging system (Vilber Lourmat). For quantification, signal intensity was analyzed using ImageJ software.

### Nitrite/nitrate quantification

The measurements were carried out as described previously [69]. In brief, a protease inhibitor mixture containing 4-(2-Aminoethyl)benzenesulfonyl fluoride (1 mg/mL), antipain, aprotinin, benzamidine, leupeptin, and pepstatin A, all at a concentration of 10 μg/mL was added to tissue and homogenized at 4 °C using a Precellys tissue homogenizer (Bertin Instruments, Montigny-le-Bretonneux, France). Followed by a centrifugation step at 10,000×*g*, 5 min, 4 °C. The supernatant was purified and concentrated using centrifugal filters (Sartorius, Göttingen, Germany; catalog #VS0192). The eluate was stored at − 80 °C until further use. Urine samples were used directly, with no prior processing steps. The amounts of nitrite and nitrate in the liquid samples were measured using an ozone-based chemiluminescence analyzer (NOA 280i, Analytix, Boldon, UK) as per previous publications [70].

### Flow cytometry analysis

Blood samples were collected in EDTA tubes and diluted 1:1 with PBS. 1 µL of a CD16/CD32 monoclonal antibody mix (Thermo Fisher Scientific, Waltham, MA, USA) was added to block non-specific Fc domains to minimize false-positive results**.** The following anti-mouse antibodies were used to stain the cells in accordance with the manufacturer’s instructions: anti-CD3 (clone 17A2; FITC; Invitrogen, Waltham, MA), anti-CD11b (clone M1/70; PerCP-Cy5.5; Invitrogen), anti-CD11c (clone HL3; BV510; BD Horizon, Franklin Lakes, NJ), anti-CD19 (clone eBio1D3; APC-Cy7; Invitrogen), anti-CD45 (clone 30-F11; eFlour450; Invitrogen), anti-Ly6C (clone HK1.4; APC; eBioscience, San Diego, CA), and anti-Ly6G (clone RB6-8C5; PE-Cy7; eBioscience. For analysis of adhesion molecule expression on myeloid cells blood samples were diluted 1:1 with PBS. 1 µL of a CD16/CD32 monoclonal antibody was added to block non-specific Fc domains to minimize false-positive results. Equal volumes of blood were distributed into four round-bottom tubes. The following anti-mouse antibodies were used to stain the cells in accordance with the manufacturer´s instructions: anti-CD45 (clone 30-F11; eFlour450; Invitrogen), anti-CD11b (clone M1/70; APC-Cy7; Invitrogen), anti-PSGL-1 (clone 4RA10; PE; Invitrogen), anti-CD49d (clone R1-2; FITC; BioLegend), anti-L-selectin (clone MEL-14; Cy5.5; BioLegend), anti-CD18 (clone H155-78; APC; BioLegend), anti-CD3 (clone 17A2; PE-Cy7; BioLegend), anti-CD19 (clone 6D5; PE-Cy7; BioLegend), anti-Ly6C (clone HK1.4; PE-Cy7; BioLegend), and anti-Ly6G (clone 1A8-Ly6g; PE-Cy7; BD Pharmingen). The cells were measured using a FACSCalibur flow cytometer (Becton Dickinson) and analyzed using FlowJo v10.7 (Becton Dickinson).

### Quantitative real-time PCR (qRT-PCR)

Total RNA was purified from the brain tissues using the RNeasy Mini Kit (Qiagen, Hilden, Germany), and 1 µg/µl RNA was used to synthesize cDNA using the Omniscript Reverse Transcription Kit Quick-Start (Qiagen Hilden, Germany). The relative expression of adhesion molecules and cytokines was measured using a LightCycler 480 II (Roche Life Sciences, Penzberg, Germany) using the following primers (all obtained from Metabion International, Planegg, Germany):

**Table:** 

Target gene	Sequence (5' to 3')	Sequence (5' to 3')
*Icam-1*	CAA TTT CTC ATG CCG CAC AG	AGC TGG AAG ATC GAA AGT CCG
*Vcam-1*	TCT TAC CTG TGC GCT GTG AC	ACT GGA TCT TCA GGG AAT GAG T
*SELE*	CCG TCC CTT GGT AGT TGC A	CAA GTA GAG CAA TGA GGA CGA TGT
*Gapdh*	ATT GTC AGC AAT GCA TCC TG	ATG GAC TGT GGT CAT GAG CC
*IL1B*	AGT GAC GGA CCC CAA AAG	AGC TGG ATG CTC TCA TCA GG
*TNF*	TCT TCT CAT TCC TGC TTG TGG	GGT CTG GGC CAT AGA ACT GA
*IL6*	GCT ACC AAA CTG GAT ATA ATC AGG A	CCA GG AGC TAT GGT ACT CCA GAA
*SELP*	GCC ATT CAG TGT GCT GAC TC	CGG AAA CTC TGG ACA TTG C
*MMP9*	AGA CGA CAT AGA CGG CAT CC	TCG GCT GTG GTT CAG TTG T
*GUCY1A1*	TCT CCC TGG TAT CAT TAA AGC GG	CAC AAA CTC GGT ACA GTC ACT TC
*GUCY1B1*	TGC TGG TGA TCC GCA ATT ATG	GGT TGA GGA CTT TGC TTG CAG
*PRKG1A*	GAC AGC GAC CGT CAA GAC TC	GAG GAT TTC ATC AGG AAG GCT C
*PRKG1B*	ACC CTG CGG GAT TTA CAG TAT	CGT CCT TCT GAT CCA ACT CCA
*NOS3*	CGA AGC GTG TGA AGG CAA C	TTG TAC GGG CCT GAC ATT TCC
*NOS1*	CTG GTG AAG GAA CGG GTC AG	CCG ATC ATT GAC GGC GAG AAT

The mRNA levels were normalized to *Gapdh* using the 2^−ΔΔCt^ method and are expressed relative to the contralateral hemisphere.

### Statistical analysis

GraphPad Prism 9 software (GraphPad Software Inc., San Diego, CA) was used to perform all statistical analyses. Unless stated otherwise, summary data are expressed as the mean ± the standard deviation (SD). All data were first tested for normality; normally distributed data were analyzed using a one-way ANOVA, while non-normally distributed data were analyzed using the Kruskal–Wallis test. Differences between groups were considered significantly different at *p* < 0.05.

### Supplementary Information

**Additional file 1: Figure S1.** Distribution of adhered Leukocytes 4 h after MCAo. A) Representative image of cerebral vasculature (cyan) and B) quantification of leukocyte adhesion from the cortical surface to a depth of 400 µm (scale bar = 50 µm). n = 5–10 per group.

**Additional file 2: Figure S2.** Physiological parameters during in vivo imaging. A) Venous blood flow velocity was measured in vivo by performing a line scan and dividing the traveled distance of erythrocytes (µm) by the respective traveled time (msec.). B) Diameter of venules and capillaries was measured in a randomly chosen vessel from the 3D two-photon images with ImageJ. C) Vital parameters were measured and monitored throughout the entire surgery via LabChart software. D) Blood gas values after imaging. n = 5–10 per group.

## Data Availability

The dataset(s) will supporting the conclusions of this article is (are) available in the Open Science Foundation repository, 10.17605/OSF.IO/XQB3Z.
