# Supplementary figures and images for: Inhaled nitric oxide suppresses neuroinflammation in experimental ischemic stroke
Source: J Neuroinflammation. 2023 Dec 15;20:301. doi: 10.1186/s12974-023-02988-3 (PMC10725028; doi:10.1186/s12974-023-02988-3)

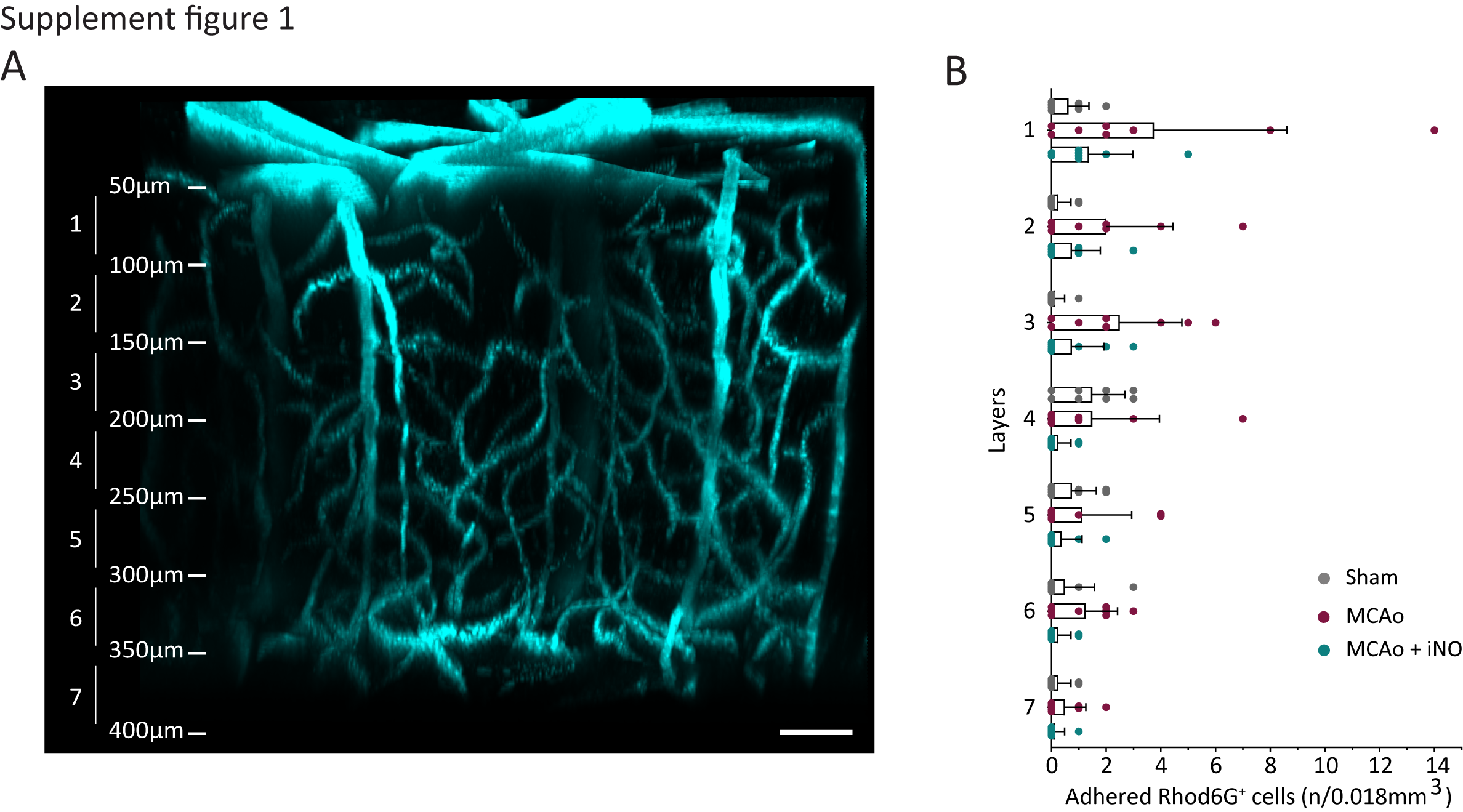

Supplement: Supplementary file 1 — Additional file 1: Figure S1. Distribution of adhered Leukocytes 4 h after MCAo. A) Representative image of cerebral vasculature (cyan) and B) quantification of leukocyte adhesion from the cortical surface to a depth of 400 µm (scale bar = 50 µm). n = 5–10 per group. [file 12974_2023_2988_MOESM1_ESM.tif]

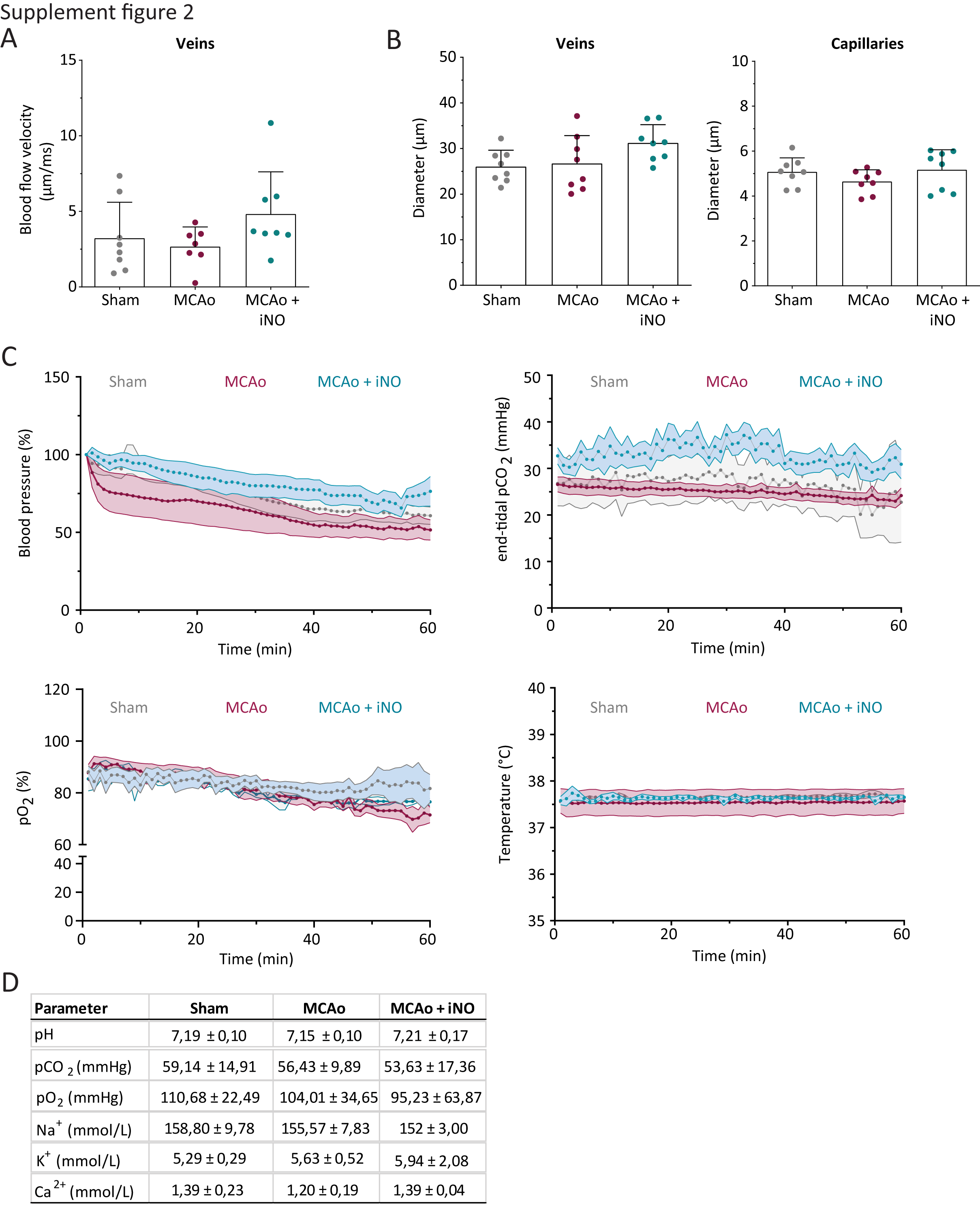

Supplement: Supplementary file 2 — Additional file 2: Figure S2. Physiological parameters during in vivo imaging. A) Venous blood flow velocity was measured in vivo by performing a line scan and dividing the traveled distance of erythrocytes (µm) by the respective traveled time (msec.). B) Diameter of venules and capillaries was measured in a randomly chosen vessel from the 3D two-photon images with ImageJ. C) Vital parameters were measured and monitored throughout the entire surgery via LabChart software. D) Blood gas values after imaging. n = 5–10 per group. [file 12974_2023_2988_MOESM2_ESM.tif]
